# *HLA-B* allele frequencies and implications for pharmacogenetics in the Kuwaiti population

**DOI:** 10.3389/fphar.2024.1423636

**Published:** 2024-10-11

**Authors:** Mohammed Dashti, Md Zubbair Malik, Abdullah Al-Matrouk, Saeeda Bhatti, Rasheeba Nizam, Sindhu Jacob, Fahd Al-Mulla, Thangavel Alphonse Thanaraj

**Affiliations:** ^1^ Genetics and Bioinformatics Department, Dasman Diabetes Institute, Kuwait City, Kuwait; ^2^ Narcotic and Psychotropic Department, Ministry of Interior, Farwaniya, Kuwait; ^3^ College of Medical Veterinary and Life Sciences, University of Glasgow, Glasgow, United Kingdom

**Keywords:** HLA-B alleles, pharmacogenetics, NGS-HLA typing, Kuwaiti population, precision medicine

## Abstract

**Objective:** This study explores the frequency of human leukocyte antigen (HLA) genes, particularly *HLA-B* alleles, within the Kuwaiti population. We aim to identify alleles with known associations to adverse drug reactions (ADRs) based on existing literature. We focus on the *HLA-B* gene due to its well-documented associations with severe cutaneous adverse reactions and the extensive pharmacogenetic research supporting its clinical relevance.

**Methods:**

We utilized the HLA-HD tool to extract, annotate, and analyse *HLA-B* alleles from the exome data of 561 Kuwaiti individuals, sequenced on the Illumina HiSeq platform. HLA typing was conducted using the HLA-HD tool with a reference panel from the IPD-IMGT/HLA database. The major *HLA-B* pharmacogenetic markers were obtained from the HLA Adverse Drug Reaction Database, focusing on alleles with significant ADR associations in published literature.

**Results:**

The distribution of *HLA-B* alleles in the Kuwaiti population revealed that the most frequent alleles were HLA-B*50:01 (10.52%), HLA-B*51:01 (9.89%), HLA-B*08:01 (6.06%), HLA-B*52:01 (4.55%), HLA-B*18:01 (3.92%), and HLA-B*41:01 (3.65%). Notably, alleles HLA-B*13:01, HLA-B*13:02, HLA-B*15:02, HLA-B*15:13, HLA-B*35:02, HLA-B*35:05, HLA-B*38:01, HLA-B*40:02, HLA-B*44:03, HLA-B*51:01, HLA-B*57:01 and HLA-B*58:01 were identified with known associations to various ADRs. For example, HLA-B*51:01 was associated with clindamycin, phenobarbital, and phenytoin, and was found in 18% of individuals.

**Conclusion:**

Our study enriches the regional genetic landscape by delineating *HLA-B* allele variations within Kuwait and across the Arabian Peninsula. This genetic insight, along with the identification of markers previously linked to drug hypersensitivity, provides a foundation for future pharmacogenetic research and potential personalized medicine strategies in the region.

## Introduction

Adverse drug reactions (ADRs) manifesting as hypersensitivity drug reactions are significant health concerns, often leading to hospitalizations and fatalities (Lazarou et al., 1998; Pirmohamed et al., 2004; Davies et al., 2009). These reactions, triggered by various chemicals, involve the immune system, particularly delayed hypersensitivity responses mediated by T cells (Shapiro and Shear, 1996; Pichler, 2003).

The major histocompatibility complex (MHC), located on chromosome 6, plays a crucial role in both innate and adaptive immunity due to its high degree of polymorphism and linkage disequilibrium (The MHC sequencing consortium, 1999; Mungall et al., 2003). The human leukocyte antigen (HLA) system, a part of the MHC, consists of genes inherited from both parents, which are expressed on the surface of antigen-presenting cells. HLA molecules are classified into three classes (I, II, and III) based on their gene location, function, expression patterns, and biochemical properties (Howell et al., 2010). Class I molecules (*HLA-A*, *HLA-B*, *HLA-C*) present intracellular peptides to cytotoxic T cells (CD8^+^), while class II molecules (*HLA-DPA1*, *HLA-DPB1*, *HLA-DQA1*, *HLA-DQB1*, *HLA-DRA*, *HLA-DRB1*) present exogenous peptides to helper T cells (CD4^+^) (Dendrou et al., 2018).

The *HLA-B* gene, characterized by a high frequency of polymorphisms and complex linkage disequilibrium, is particularly challenging for traditional genotyping techniques. Next-generation sequencing (NGS) offers high-throughput and accurate HLA typing, essential for studying genetic diversity and phenotypic correlations worldwide (Claeys et al., 2023). Studies have identified *HLA-B* as a key genetic factor in ADRs, particularly severe cutaneous adverse reactions (SCARs) such as Stevens-Johnson syndrome (SJS), toxic epidermal necrolysis (TEN), and drug rash with eosinophilia and systemic symptoms (DRESS) (Jantararoungtong et al., 2021; Kloypan et al., 2021). For instance, HLA-B*15:02 is linked to carbamazepine-induced SJS/TEN (Ferrell and McLeod, 2008; Chang et al., 2011; Wei et al., 2012), and HLA-B*58:01 is associated with allopurinol-induced SCARs (Gonçalo et al., 2013). Screening for these alleles before prescribing medications can significantly reduce severe reactions, underscoring the clinical utility of pharmacogenetic testing (Chen et al., 2018).

Our focus on the *HLA-B* gene is based on the extensive body of pharmacogenetic research available. According to the HLA Adverse Drug Reaction Database (HLA-ADR) on the Allele Frequency Net Database (allelefrequencies.net), *HLA-B* alleles are more extensively studied compared to *HLA-A* and *HLA-C* genes. Other studies have similarly focused on the *HLA-B* gene in various populations due to its strong associations with pharmacogenomics (Koomdee et al., 2022; Yuliwulandari et al., 2024) and immunogenetics (Sajulga et al., 2022; Darbas et al., 2023). The Arabian Peninsula populations are underrepresented in global studies, and data on *HLA-B* allele frequencies can aid in understanding drug hypersensitivity in these populations (Arnaiz-Villena et al., 2019; Jawdat et al., 2020; Alfraih et al., 2021; Hajjej et al., 2020; Albalushi et al., 2014; Dashti et al., 2022; Ameen et al., 2020).

The latest attempt to explore *HLA-B* alleles in the Kuwaiti population was conducted by Ameen et al. (2020), focusing on reporting the most frequent alleles of classical HLA class I and class II genes using low-resolution typing. The most common group of *HLA-B* alleles reported was B*50:01, with a frequency of 12% (Ameen et al., 2020). Additionally, neither the study by Ameen et al. (2020) nor other studies have explored *HLA-B* alleles as pharmacogenetic markers in Kuwait (Moussa et al., 1985; Al-Bader et al., 2019)

Therefore, this study aims to explore the frequency of *HLA-B* alleles in the Kuwaiti population using high-resolution typing and to identify alleles with known associations to ADRs based on existing literature. Our goal is to determine the prevalence of these pharmacogenetically relevant *HLA-B* alleles in Kuwait and compare our data with those from other Gulf countries, contributing to a foundational understanding that may inform future personalized medicine initiatives in the region.

In a previous study, we ranked NGS-based HLA typing tools, focusing on those that are alignment-based and utilize the genetic diversity catalogued in the IPD-IMGT/HLA database (Robinson et al., 2020) for accurate allele calling. Our ranking was based on multiple independent benchmarking studies (Chen et al., 2021; Thuesen et al., 2022; Claeys et al., 2023), where we prioritized the top tools based on their performance (Dashti et al., 2024). We then evaluated the computational efficiency and capabilities of these top HLA typing tools on whole exome sequencing (WES) data, identifying HLA-HD (Kawaguchi et al., 2017) as one of the top performers. Additionally, we compared the performance of the HLA-HD tool against clinical grade HLA typing tool using various NGS datasets, confirming its reliability and consistency across multiple HLA loci (Dashti et al., 2024).

This work provides a solid foundation for using the HLA-HD tool in our current research, ensuring that our findings are both accurate and relevant to population-scale studies of *HLA-B* allele frequencies and their potential implications for drug hypersensitivity.

## Methods and materials

### Ethics Statement

The study was approved by the Ethical Review Committee at Dasman Diabetes Institute in Kuwait, in accordance with the guidelines outlined in the Declaration of Helsinki. The project reference number is RAHM 2019-025.

### Study samples

Whole exome sequence data from 561 Kuwaiti individuals used in this study were sequenced on the Illumina HiSeq platform using the TruSeq Exome Enrichment kit and the Nextera Rapid Capture Exome kit (Illumina Inc., United States). A total of 561 Kuwaitis, including 271 males and 290 females, with an average age of 52 years, participated in the study. All participants provided informed consent prior to recruitment. These samples are part of an ongoing project on the Kuwaiti population aimed at capturing the extent of exome variation within the population, involving a larger cohort than previously reported (John et al., 2018). All participants were healthy and free of Mendelian or rare genetic disorders. For more details about the sequencing protocol used in the initial phase of this project, please refer to John et al., 2018.

### HLA-B typing

Raw sequencing data in BCL format obtained from the Illumina sequencing platform were converted to Fastq format using the bcl2fastq v2.20 Conversion Software (Illumina, United States). The converted raw paired-end reads of 561 Kuwaiti individuals were then processed with the HLA-HD tool version 1.4.0 (Kawaguchi et al., 2017) to determine the *HLA-B* alleles. This was achieved by mapping the reads to the relevant region of the human genome reference using Bowtie 2 tool version 2.5.2 (Langmead and Salzberg, 2012). A comprehensive reference panel from the IPD-IMGT/HLA database version 3.46 (accessible at http://hla.alleles.org and https://www.ebi.ac.uk/ipd/imgt/hla/licence/) (Robinson et al., 2020) was used for genomic imputation, and a score based on weighted read counts was calculated to select the most suitable pair of alleles.

### HLA-B pharmacogenomic markers

The major *HLA-B* pharmacogenetic markers were obtained from the HLA Adverse Drug Reaction Database website (http://www.allelefrequencies.net/) using a *p*-value filter of <0.01 across all ethnicities (accessed on 15 July 2024). Given that the database is continually updated by researchers, a comprehensive manual review was performed to identify relevant markers. This review aimed to confirm the association of each marker with drug hypersensitivity, ensuring they met the criteria of being risk alleles, having passed multivariate analysis with significant adjusted *p*-values, and being correctly typed. This process resulted in the identification of 17 unique *HLA-B* alleles associated with pharmacogenetic risk: HLA-B*13:01, HLA-B*13:02, HLA-B*15:02, HLA-B*15:11, HLA-B*15:13, HLA-B*15:27, HLA-B*35:02, HLA-B*35:05, HLA-B*38:01, HLA-B*39:05, HLA-B*40:02, HLA-B*44:03, HLA-B*51:01, HLA-B*57:01, HLA-B*58:01, HLA-B*58:05, and HLA-B*59:01.

### Comparison of HLA-B top alleles with Arab Gulf countries and other ethnic groups

In addition to analysing the *HLA-B* allele frequencies within the Kuwaiti population, we compared these frequencies with those reported in other Arab Gulf countries and in various continental ethnic groups.

For the regional comparison, we utilized published literature on *HLA-B* alleles in Gulf countries, including Saudi Arabia (Jawdat et al., 2020), Qatar (Dashti et al., 2022), Bahrain (Hajjej et al., 2020), the United Arab Emirates (Arnaiz-Villena et al., 2019), and Oman (Albalushi et al., 2014). We extracted and compared the 10 most frequent *HLA-B* alleles in each population with the top 10 most frequent *HLA-B* alleles identified in the Kuwaiti population.

For the broader comparison with other ethnic groups, we utilized the Allele Frequency Net Database (accessed on 13 August 2024). This database provides comprehensive allele frequency data from a variety of ethnic groups. We queried the top 10 frequent *HLA-B* alleles in the Kuwaiti population and compared them with those in regions such as Europe, North Africa, North America, South Asia, Western Asia, and Sub-Saharan Africa. The data sources were filtered based on literature, and the study type was set to anthropology. We sorted the studies based on cohort size, selecting the most representative studies for each region. In cases where a specific allele was not investigated in the primary study, we used the next best study by cohort size for our comparative analysis.

### Statistical analysis

The *HLA-B* allele frequencies were calculated by manually counting the occurrences of each allele and dividing them by the total number of *HLA-B* alleles in the cohort. For a diploid cohort, this total is twice the number of individuals, as each individual has two *HLA-B* alleles.

To assess the deviation from Hardy-Weinberg equilibrium (HWE), we utilized the R software, version 3.6.2 (R Core Team, 2023). The observed genotype frequencies were compared to the expected frequencies under HWE assumptions. Expected genotype counts were estimated based on the observed allele frequencies, while the actual genotype counts represented the genotypes observed in the cohort. Genotype frequencies were calculated as the proportion of each genotype among the total number of observed genotypes. The significance of the deviation from HWE was evaluated using *p*-values, with a threshold of *p* < 0.05 indicating significant deviation.

For the comparison of the most frequent *HLA-B* alleles in the Kuwaiti population with those in other ethnic groups, we calculated 95% confidence intervals using the R software. The confidence intervals were derived based on the extracted allele frequencies and the corresponding sample sizes of each ethnic group. This statistical approach allowed us to determine the range within which the true allele frequency is likely to fall, with a 95% level of confidence. Comparison of allele frequencies, along with their confidence intervals, was then visualized using stacked bar chart generated in R, facilitating the assessment of genetic similarities and differences across the regions.

## Results

### HLA-B allele frequencies

In total, we identified 160 unique *HLA-B* alleles in our study of 561 Kuwaiti individuals (Supplementary Table S1). All the identified *HLA-B* alleles observed in more than one individual (n > 1) are presented in Table 1. The frequency of the observed 143 distinct *HLA-B* alleles among the 561 Kuwaiti individuals is listed in Table 1. The most frequent *HLA-B* alleles identified were HLA-B*50:01 (10.52%), HLA-B*51:01 (9.89%), HLA-B*08:01 (6.06%), HLA-B*52:01 (4.55%), HLA-B*18:01 (3.92%), and HLA-B*41:01 (3.65%). The *HLA-B* alleles passed the quality control for HWE >10^−3^.

**TABLE 1:** Observed *HLA-B* alleles (n > 1) in Kuwaiti population.

*HLA-B* alleles	No. of alleles	Allele frequency (%)	Estimated genotype count^a^	No. of observed count of genotypes^b^	Genotype frequency (%)^c^	HW *p*-value
B*50:01	118	10.52	6.2	8	1.43	0.79
B*51:01	111	9.89	5.49	10	1.78	0.3
B*08:01	68	6.06	2.06	1	0.18	1
B*52:01	51	4.55	1.16	1	0.18	0.48
B*18:01	44	3.92	0.86	2	0.36	1
B*41:01	41	3.65	0.75	0	0	1
B*35:03	35	3.12	0.55	3	0.53	0.62
B*35:08	33	2.94	0.49	2	0.36	1
B*49:01	32	2.85	0.46	0	0	1
B*07:02	32	2.85	0.46	2	0.36	1
B*35:02	29	2.58	0.37	1	0.18	1
B*14:02	28	2.5	0.35	1	0.18	1
B*40:06	27	2.41	0.32	2	0.36	1
B*35:01	26	2.32	0.3	2	0.36	1
B*15:17	25	2.23	0.28	0	0	1
B*53:01	25	2.23	0.28	0	0	1
B*13:02	25	2.23	0.28	0	0	1
B*58:01	23	2.05	0.24	0	0	1
B*38:01	22	1.96	0.22	1	0.18	1
B*44:03	15	1.34	0.1	0	0	1
B*15:03	14	1.25	0.09	0	0	1
B*57:01	12	1.07	0.06	0	0	1
B*73:01	11	0.98	0.05	1	0.18	1
B*42:01	11	0.98	0.05	0	0	1
B*55:01	11	0.98	0.05	0	0	1
B*15:10	10	0.89	0.04	0	0	1
B*45:01	10	0.89	0.04	0	0	1
B*07:05	9	0.8	0.04	0	0	1
B*44:02	8	0.71	0.03	0	0	1
B*15:220	8	0.71	0.03	0	0	1
B*41:02	7	0.62	0.02	0	0	1
B*51:08	7	0.62	0.02	0	0	1
B*40:01	7	0.62	0.02	0	0	1
B*39:01	6	0.53	0.02	0	0	1
B*42:02	6	0.53	0.02	0	0	1
B*15:08	6	0.53	0.02	0	0	1
B*39:24	5	0.45	0.01	0	0	1
B*58:02	5	0.45	0.01	0	0	1
B*15:16	5	0.45	0.01	1	0.18	1
B*50:57	4	0.36	0.01	0	0	1
B*14:01	4	0.36	0.01	1	0.18	1
B*37:01	4	0.36	0.01	1	0.18	1
B*40:02	4	0.36	0.01	0	0	1
B*78:02	4	0.36	0.01	0	0	1
B*57:03	4	0.36	0.01	0	0	1
B*40:12	3	0.27	0	0	0	1
B*27:02	3	0.27	0	0	0	1
B*44:05	3	0.27	0	0	0	1
B*51:237	3	0.27	0	0	0	1
B*47:03	3	0.27	0	0	0	1
B*47:01	2	0.18	0	0	0	1
B*27:03	2	0.18	0	0	0	1
B*39:06	2	0.18	0	0	0	1
B*18:03	2	0.18	0	0	0	1
B*13:01	2	0.18	0	0	0	1
B*14:03	2	0.18	0	0	0	1
B*15:09	2	0.18	0	0	0	1
B*51:02	2	0.18	0	0	0	1
B*57:02	2	0.18	0	0	0	1
B*58:08	2	0.18	0	0	0	1
B*44:09	2	0.18	0	0	0	1
B*35:516	2	0.18	0	0	0	1
B*51:151	2	0.18	0	1	0.18	1
B*51:285	2	0.18	0	0	0	1
B*15:01	2	0.18	0	0	0	1
B*15:18	2	0.18	0	0	0	1
B*07:06	2	0.18	0	0	0	1
B*27:05	2	0.18	0	0	0	1
B*39:10	2	0.18	0	0	0	1
B*27:07	2	0.18	0	0	0	1
B*15:29	2	0.18	0	0	0	1
B*13:136	2	0.18	0	0	0	1

^a^ Estimatedgenotype count refers to the expected number of genotypes based on allele frequencies.

^b^ Observed count is the actual number of genotypes observed in the cohort.

^c^ Genotype frequency is the proportion of the genotype among the total genotypes.

### HLA-B genotype frequencies

Examining the *HLA-B* genotypes of 561 Kuwaiti individuals revealed 370 distinct genotypes in total. The most frequently observed genotype among the population, as listed in Table 2, was B*50:01 + B*51:01, which was the most common at a rate of 3.57%. The frequencies of the rest of the frequent genotypes were under 3% in the Kuwaiti population.

**TABLE 2:** Top 10 observed *HLA-B* genotypes in Kuwaiti population.

*HLA-B* genotypes	No of individuals	Frequency (%)
B*50:01 + B*51:01	20	3.57
B*51:01 + B*51:01	10	1.78
B*08:01 + B*51:01	9	1.60
B*50:01 + B*50:01	8	1.43
B*08:01 + B*50:01	6	1.07
B*35:03 + B*50:01	6	1.07
B*51:01 + B*52:01	5	0.89
B*50:01 + B*52:01	5	0.89
B*41:01 + B*51:01	5	0.89
B*14:02 + B*50:01	5	0.89

### Prevalence of HLA-B pharmacogenomic markers in the Kuwaiti population

We identified twelve *HLA-B* pharmacogenetic markers that associated with ADRs in 235 of the 561 Kuwaiti individuals (41.1%) (Table 3). The most prevalent pharmacogenetic markers were HLA-B*51:01, found in 18% of individuals and associated with phenytoin, phenobarbital, carbamazepine, and clindamycin (Niihara et al., 2012; Kaniwa et al., 2013; Manuyakorn et al., 2020; John et al., 2021), HLA-B*35:02, present in 5% and associated with minocycline (Urban et al., 2017), HLA-B*13:02, present in 4.5% and associated with allopurinol, lamotrigine, and oxcarbazepine (He et al., 2012; Kim et al., 2017; Wu et al., 2018), and HLA-B*58:01, present in 4.1% and associated with allopurinol (Lonjou et al., 2008; Gonçalo et al., 2013; Sukasem et al., 2016; Fontana et al., 2021). Other identified markers included HLA-B*38:01 (3.9%), associated with lamotrigine and other aromatic antiepileptic drugs (Ramírez et al., 2017), HLA-B*44:03 (2.7%), associated with phenytoin (Ueta et al., 2014; Park et al., 2016; Wakamatsu et al., 2021), HLA-B*57:01 (2.1%), associated with abacavir (Mallal et al., 2002), HLA-B*40:02 (0.7%), associated with oxcarbazepine (Moon et al., 2016), HLA-B*13:01 (0.4%), associated with dapsone, salazosulfapyridine, and phenytoin (Yang et al., 2014; Wu et al., 2018; Su et al., 2019; Ahmed et al., 2021), HLA-B*15:02 (0.2%), associated with carbamazepine and phenytoin (Ferrell and McLeod, 2008; Chang et al., 2011; Wei et al., 2012; Ahmed et al., 2021), HLA-B*15:13 (0.2%), associated with phenytoin (Chang et al., 2017), and HLA-B*35:05 (0.2%), associated with nevirapine (Ahmed et al., 2021).

**TABLE 3:** Major *HLA-B* pharmacogenetics markers and genotypes in Kuwaiti population.

Associated Drug(s)^a^	Pharmacogenetic marker	Genotype	Individuals	Percentage of cohort (number of individuals)
Clindamycin, Phenobarbital, Phenytoin	HLA-B*51:01	Homozygous	10	18% (101/561)
		Heterozygous	91	
Minocycline	HLA-B*35:02	Homozygous	1	5% (28/561)
		Heterozygous	27	
Allopurinol, Lamotrigine, Oxcarbazepine	HLA-B*13:02	Homozygous	0	4.5% (25/561)
		Heterozygous	25	
Allopurinol	HLA-B*58:01	Homozygous	0	4.1% (23/561)
		Heterozygous	23	
Lamotrigine, Antiepileptic drugs	HLA-B*38:01	Homozygous	1	3.9% (22/561)
		Heterozygous	21	
Dipyrone, Phenytoin, Ticlopidine	HLA-B*44:03	Homozygous	0	2.7% (15/561)
		Heterozygous	15	
Abacavir, Carbamazepine, Flucloxacillin, Lamotrigine	HLA-B*57:01	Homozygous	0	2.1% (12/561)
		Heterozygous	12	
Oxcarbazepine	HLA-B*40:02	Homozygous	0	0.7% (4/561)
		Heterozygous	4	
Dapsone, Lamotrigine, Phenobarbital, Phenytoin, Salazosulfa-Pyridine, Sulfasalazine, Trichloroethylene	HLA-B*13:01	Homozygous	0	0.4% (2/561)
		Heterozygous	2	
Carbamazepine, Oxcarbazepine, Phenytoin, Sulfamethoxazole	HLA-B*15:02	Homozygous	0	0.2% (1/561)
		Heterozygous	1	
Phenytoin	HLA-B*15:13	Homozygous	0	0.2% (1/561)
		Heterozygous	1	
Benznidazole, Nevirapine	HLA-B*35:05	Homozygous	0	0.2% (1/561)
		Heterozygous	1	
Total				41.1% (235/561)

^a^ Drugs assoiated with HLA, alleles were obtained from the HLA, Adverse Drug Reaction Database (HLA-ADR) on the Allele Frequency Net Database (allelefrequencies.net).

### Comparison of HLA-B top alleles across Arab Gulf countries and other regions

Table 4 presents a comparative analysis of the top 10 most frequent *HLA-B* alleles in the Kuwaiti population with those observed in other Arab Gulf countries, including Saudi Arabia, Qatar, Bahrain, the United Arab Emirates, and Oman. This comparison highlights the similarities and differences in *HLA-B* allele distribution across these closely related regions. The data indicate that many of the most prevalent *HLA-B* alleles in the Kuwaiti population are also commonly found in neighbouring Gulf countries, suggesting shared genetic backgrounds and potential regional influences on allele frequencies.

**TABLE 4:** Top 10 frequent *HLA-B* alleles, as ordered considering allele frequency (AF), in the Arab populations from the Gulf region.

Kuwait		Saudi Arabia		Qatar		Bahrain		United Emirates		Oman	
*HLA-B*	AF (%)	*HLA-B*	AF (%)	*HLA-B*	AF (%)	*HLA-B*	AF (%)	*HLA-B*	AF (%)	*HLA-B*	AF (%)
B*50:01:01	10.25	B*51:01:01	19	B*50:01:01	18.21	B*35:01:02	12.9	B*50:01	14.42	B*35	15.3
B*51:01:01	9.71	B*50:01:01	12.4	B*51:01:01	17.35	B*47:01:01	7.1	B*51:01	13.46	B*51	14.7
B*08:01:01	5.97	B*08:01:01	6.9	B*08:01:01	7.24	B*44:02:03	6.9	B*52:01	5.77	B*08	9.3
B*52:01:01	4.46	B*07:02:01	5	B*07:02:01	4.60	B*18:01:01	6.6	B*15:17	4.81	B*58	9.1
B*41:01:01	3.65	B*53:01:01	3.9	B*40:06:01	4.28	B*15:10	6	B*44:03	4.81	B*40	6.4
B*18:01:01	3.65	B*41:01	3.4	B*58:01:01	3.42	B*58:01:01	6	B*58:01	4.81	B*52	6
B*35:03:01	3.12	B*58:01:01	3.4	B*49:01:01	2.82	B*52:01:01	5.4	B*35:01	3.85	B*15	6
B*35:08:01	2.94	B*35:01:01	2.8	B*18:01:01	2.78	B*51:02:01	5.1	B*35:02	2.88	B*18	4.2
B*49:01:01	2.85	B*18:01:01	2.7	B*53:01:01	2.69	B*08:01:01	4.3	B*40:06	2.88	B*50	4.2
B*07:02:01	2.67	B*49:01:01	2.5	B*35:01:01	2.55	B*42:01:01	4.3	B*58:02	2.88	B*07	3.1
This study		Jawdat et al. (2020)		Dashti et al. (2022)		Hajjej et al. (2020)		Arnaiz-Villena et al. (2019)		Albalushi et al. (2014)	

Note: Alleles in *italics* are also recognized pharmacogenetic markers associated with adverse drug reactions.

**FIGURE 1:**
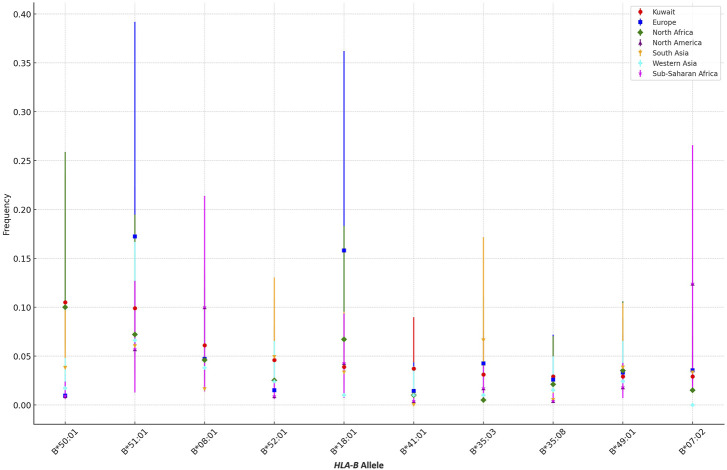
Comparison of the Top 10 *HLA-B* Allele Frequencies in the Kuwaiti Population Across Different Ethnic Groups. A comparative analysis of the top 10 most frequent *HLA-B* alleles in the Kuwaiti region and various other regions, including Europe, North Africa, North America, South Asia, Western Asia, and Sub-Saharan Africa. The frequencies of these alleles are presented with 95% confidence intervals to highlight genetic similarities and differences.

Expanding beyond the Gulf region, Figure 1 illustrates the differences in the frequencies of the top *HLA-B* alleles between the Kuwaiti population and various other ethnic groups, including populations from Europe, North Africa, North America, South Asia, Western Asia, and Sub-Saharan Africa.

The analysis reveals that certain *HLA-B* alleles in the Kuwaiti population, such as B*50:01 and B*18:01, are more closely aligned with frequencies observed in other Middle Eastern regions, including Western Asia. However, these alleles show significant differences when compared to populations from Europe and North America, where these alleles are much less common. For instance, B*50:01, with a frequency of 0.105 in Kuwait, is almost absent in European (0.0094) and North American (0.009) populations, as indicated by non-overlapping confidence intervals, suggesting a distinct genetic profile in these regions.

Conversely, alleles like B*07:02, which is present in the Kuwaiti population, show more similarity in frequency with North African and Sub-Saharan African populations, indicating a shared genetic background or historical gene flow between these regions. In contrast, alleles such as B*51:01 demonstrate variability across all regions, with Kuwait showing closer frequencies to South Asia and Western Asia compared to other regions.

## Discussion

In total, we identified 160 unique *HLA-B* alleles at high resolution in our study of 561 Kuwaiti individuals. High-resolution typing can be beneficial for pharmacogenetic studies, as it has the potential to increase the statistical power and accuracy in associating specific alleles with diseases and ADRs. This higher level of detail may help to better understand the variability in drug responses among individuals. Recent studies suggest that some synonymous variants, while not altering the protein sequence, may still impact splicing, RNA stability, RNA folding, translation, or co-translational protein folding, and could be implicated in various human diseases (Lin et al., 2023; Sharma et al., 2019). However, the risk of manifesting an ADR is also likely influenced by a combination of genetic factors, such as specific HLA alleles, and environmental variables, reflecting a multifactorial nature to these outcomes.

The most frequent alleles identified were HLA-B*50:01 (10.52%), HLA-B*51:01 (9.89%), HLA-B*08:01 (6.06%), HLA-B*52:01 (4.55%), HLA-B*18:01 (3.92%), and HLA-B*41:01 (3.65%). These findings align with the previously reported distribution of the most frequent *HLA-B* alleles in Kuwait (Ameen et al., 2020), which focused on the top alleles within the classical HLA class I and class II genes. Our study examines all *HLA-B* alleles present in our cohort, with particular emphasis on their relevance as pharmacogenetic markers. This demonstrates that HLA typing using WES data can effectively capture the same allele frequencies identified by the combination of sequence-specific oligonucleotide (SSO) probe-based hybridization and high-resolution HLA genotyping, as employed by Ameen et al. (2020).

Furthermore, twelve *HLA-B* pharmacogenomic markers were identified in 235 of the 561 (41.1%) Kuwaiti individuals. The most frequent marker, accounting for 18% of the Kuwaiti individuals, is HLA-B*51:01. This allele has been previously reported to be involved in the pathogenesis of SJS/TEN associated with phenobarbital (an antiepileptic drug used to control seizures) in the Japanese population (Kaniwa et al., 2013), phenytoin (another antiepileptic drug) in the South Indian Tamil (John et al., 2021) and Thai (Tassaneeyakul et al., 2016; Manuyakorn et al., 2020) populations, carbamazepine (an anticonvulsant and mood stabilizer) in the Japanese population (Niihara et al., 2012), and clindamycin (an antibiotic) in the Han Chinese population (Yang et al., 2017). Therefore, HLA-B*51:01 may serve as a susceptibility factor for SJS/TEN in Asian populations. Our previous study also revealed that HLA-B*51:01 is the most frequent pharmacogenetic *HLA-B* marker, carried by 26.67% of Qatari individuals (Dashti et al., 2022). Additionally, the allele frequency of HLA-B*51:01 has been shown to be high in other Arab Gulf countries (Table 4). These similarities in allele frequencies may be due to a shared gene pool, potentially influenced by historical migrations, geographic proximity, and common ancestry among the Gulf Cooperation Council (GCC) countries.

The second most frequent pharmacogenetic marker identified is HLA-B*35:02, which is carried by 5% of the studied Kuwaiti individuals. This allele has been associated with minocycline (an antibiotic commonly used to treat bacterial infections)-induced drug-induced liver injury in a Caucasian cohort in the United States (Urban et al., 2017). In the Qatari population, the allele frequency of HLA-B*35:02 is 1.59% (Dashti et al., 2022), and in the Emirati population, it is 2.88% (Arnaiz-Villena et al., 2019), which is very similar to the Kuwaiti population’s allele frequency of 2.58%.

HLA-B*13:02 allele is the third most prevalent pharmacogenetic marker, at 4.5%, in the Kuwaiti cohort. The allele frequency of this marker is higher in the Kuwaiti population compared to other Arab populations in the Gulf region, where it is less than 1% in Qatar and not among the top ten *HLA-B* alleles in the Arabs of the Gulf countries (Table 4). HLA-B*13:02 allele has been nominally associated with lamotrigine (an antiepileptic drug used to treat epilepsy and bipolar disorder)-induced SCAR in the Korean population (Kim et al., 2017). It is also a marker for oxcarbazepine (an antiepileptic drug used to treat partial seizures)-induced maculopapular eruption in the Southern Han Chinese population (He et al., 2012). Additionally, HLA-B*13:02 has been associated with allopurinol (a medication used to treat gout and hyperuricemia)-induced DRESS in the Shanghai population (Wu et al., 2018).

The fourth prevalent pharmacogenetic marker is HLA-B*58:01, which is carried by 4.1% of the Kuwaiti cohort. Upon examining the frequencies of this allele in neighbouring countries (Table 4), we found that HLA-B*58:01 is among the top ten most frequent alleles in the Qatari, Saudi, Bahraini, and Emirati populations. The same can be said for Oman; however, the available HLA typing in Oman was conducted at low resolution, where HLA-B*58 is frequent. Nevertheless, higher resolution typing is needed to confirm the exact allele. Pharmacogenetic studies have demonstrated an association between HLA-B*58:01 and allopurinol-induced SCARs across diverse ethnicities, including African American (Fontana et al., 2021), European (Lonjou et al., 2008; Gonçalo et al., 2013), and Asian (Sukasem et al., 2016) populations. The risk of allopurinol-induced SCARs is associated with a gene dosage effect of HLA-B*58:01 on renal function (Chung et al., 2015; Ng et al., 2016), as well as with increased plasma levels of the allopurinol metabolite (Ng et al., 2016). Allopurinol is used to lower blood uric acid levels induced by chemotherapy and to prevent the formation of certain types of kidney stones (Jung et al., 2015). Nevertheless, it has been suggested that additional genetic variations beyond the HLA region might also contribute to the risk (Tohkin et al., 2013).

The fifth most prevalent pharmacogenetic marker in our study is HLA-B*38:01, which is associated with SCARs induced by lamotrigine and phenytoin in the Spanish Caucasian population (Ramírez et al., 2017). This allele is carried by 3.9% of the Kuwaiti cohort. However, it has a low allele frequency in the Qatari population (Dashti et al., 2022) and is not among the top frequent alleles in Arab populations from the Gulf region (Table 4).

Another pharmacogenetic marker identified is HLA-B*44:03, which is carried by 2.7% of the Kuwaiti cohort, a percentage similar to that observed in the Qatari population (2.8%) (Dashti et al., 2022), and is very frequent in the Emirati population (Arnaiz-Villena et al., 2019). This allele has been associated with cold-medicine (multi-ingredient cold and anti-inflammatory drug remedies)-induced SJS/TEN in Japanese (Ueta et al., 2014) and Brazilian (Wakamatsu et al., 2021) populations. Additionally, another study suggests a potential correlation between HLA-B*44:03 and lamotrigine-induced SJS/TEN in Koreans (Park et al., 2016).

In addition, the HLA-B*57:01 pharmacogenetic marker is carried by 2.1% of the Kuwaiti individuals in this study. This allele is also present at a similar percentage in the Qatari population (Dashti et al., 2022); however, it is not among the top 10 most common *HLA-B* alleles in any of the Gulf countries (Table 4). As a pharmacogenetic marker, HLA-B*57:01 is known to be associated with abacavir (an antiretroviral medication)-induced hypersensitivity (Mallal et al., 2002) and is more prevalent in Caucasian populations compared to Asian populations (Jung et al., 2018).

Moreover, we have identified several pharmacogenetic markers, each carried by less than 1% of the Kuwaiti cohort, and none are among the top frequent alleles of Arabs from the Gulf region (Table 4). Among these are the HLA-B*40:02 allele, associated with oxcarbazepine-induced maculopapular eruption in the Korean population (Moon et al., 2016), and the HLA-B*13:01 allele, linked to dapsone-induced SCARs in Thai and Han Chinese populations (Ahmed et al., 2021), salazosulfapyridine-induced drug rash with DRESS in the Shanghai and Han Chinese populations (Yang et al., 2014; Wu et al., 2018), and phenytoin-related SCARs in East Asians (Su et al., 2019). Additionally, the HLA-B*15:02 allele is known for its association with carbamazepine and phenytoin-induced SJS/TEN, particularly in Southeast Asian populations (Ferrell and McLeod, 2008; Chang et al., 2011; Wei et al., 2012; Ahmed et al., 2021), while the HLA-B*15:13 allele is associated with phenytoin-induced SCARs in the Malay population (Chang et al., 2017). Furthermore, the HLA-B*35:05 allele has been linked to nevirapine-induced hypersensitivity reactions in various ethnic groups (Ahmed et al., 2021).

In general, our data shows that the majority of the most prevalent *HLA-B* alleles in the Kuwaiti population are common in other Gulf countries (Table 4). This demonstrates that repurposing WES datasets for HLA typing to explore the frequency of *HLA* genes relevant to disease or pharmacology on a population scale is feasible. Additionally, some of the frequent *HLA-B* alleles serve as pharmacogenetic markers, indicating potential opportunities for collaborative regional health strategies to address shared pharmacogenetic risks. However, there are slight variations in the top frequent *HLA-B* alleles among these countries, reflecting genetic diversity influenced by factors such as genetic drift, selection pressure, or historical migration.

Our study highlights significant differences in the frequencies of various *HLA-B* alleles between the Kuwaiti population and other regions, underscoring the unique genetic heritage of Kuwait, particularly when compared to Europe, North America, and even neighbouring regions like Western Asia. The distinct allele frequencies observed, such as those of B*50:01 and B*18:01, reflect true population-specific patterns, as indicated by non-overlapping confidence intervals.

Incorporating other ethnic groups into the analysis further enriches our understanding of the genetic diversity across regions. For example, while alleles like B*07:02 show similar frequencies across diverse populations, others, such as B*51:01, exhibit considerable variability. These findings emphasize the influence of regional and ethnic factors in shaping *HLA-B* allele distribution, which may have important implications for disease susceptibility, transplantation compatibility, and other health-related outcomes in the Kuwaiti population.

The current study has a few limitations. First, the relatively small sample size in our investigation may have influenced the accuracy of frequency estimates for the loci examined, and there is a possibility of overlooking low-frequency alleles due to this limitation. Additionally, it is important to note that the *HLA-B* pharmacogenetic markers analysed in this study are reference markers derived from databases and studies conducted in other populations, such as those from the HLA Adverse Drug Reaction Database. These markers may be ethnicity-specific and might not be causative of ADRs in our Kuwaiti population. As a result, we may have missed additional potential markers specific to the Kuwaiti population that reflect differences in genetic backgrounds. Furthermore, this study used the HLA-HD tool for HLA typing. While benchmark studies (Chen et al., 2021; Thuesen et al., 2022; Claeys et al., 2023) have shown that other top-performing tools, such as HLA*LA (Dilthey et al., 2019) and HISAT-genotype (Kim et al., 2019), might be more consistent and accurate in typing HLA Class I genes, the difference in consistency is marginal. Additionally, some of these tools are computationally intensive, making them less suitable for population-scale projects. Importantly, the HLA-HD tool does not detect novel alleles; this limitation was known prior to the study’s design, as our aim was to analyse the distribution of known *HLA-B* alleles rather than to discover novel ones. Therefore, further studies are needed to confirm the association of the current pharmacogenetic markers with ADRs in our population and to identify additional pharmacogenetic markers that may be relevant.

## Conclusion

Our study enriches the regional genetic landscape by delineating *HLA-B* allele variations within Kuwait and across the Arabian Peninsula. This detailed characterization is invaluable for future studies on genetic diversity, disease risk, and pharmacogenetics, ultimately contributing to personalized medicine strategies in the region. By determining the frequency of pharmacogenetic markers, previously reported in different populations, within the Kuwaiti population, we provide a solid foundation for future pharmacogenetic research. While these markers are not necessarily causative of ADRs in our population, they offer valuable insights. Future research should focus on hypersensitivity studies involving different drugs and *HLA-B* alleles, as well as exploring additional *HLA* genes variations, to further advance personalized healthcare strategies in the Gulf region.

## Data Availability

The original contributions presented in the study are publicly available. This data can be found here: SRA repository, PRJNA1166832 (https://www.ncbi.nlm.nih.gov/sra/PRJNA1166832).

## Supplementary material

SUPPLEMENTARY TABLE S1 Raw HLA-B alleles identified in 561 Kuwaiti individuals.
